# Systemic physiology augmented functional near-infrared spectroscopy: a powerful approach to study the embodied human brain

**DOI:** 10.1117/1.NPh.9.3.030801

**Published:** 2022-07-11

**Authors:** Felix Scholkmann, Ilias Tachtsidis, Martin Wolf, Ursula Wolf

**Affiliations:** a University of Bern, Institute of Complementary and Integrative Medicine, Bern, Switzerland; b University Hospital Zurich, University of Zurich, Biomedical Optics Research Laboratory, Neonatology Research, Department of Neonatology, Zurich, Switzerland; c University College London, Biomedical Optics Research Laboratory, Department of Medical Physics and Biomedical Engineering, London, United Kingdom

**Keywords:** systemic physiology augmented functional near-infrared spectroscopy, functional near-infrared spectroscopy, integrative physiology, neurosystemic functional connectivity

## Abstract

In this Outlook paper, we explain why an accurate physiological interpretation of functional near-infrared spectroscopy (fNIRS) neuroimaging signals is facilitated when systemic physiological activity (e.g., cardiorespiratory and autonomic activity) is measured simultaneously by employing systemic physiology augmented functional near-infrared spectroscopy (SPA-fNIRS). The rationale for SPA-fNIRS is twofold: (i) SPA-fNIRS enables a more complete interpretation and understanding of the fNIRS signals measured at the head since they contain components originating from neurovascular coupling and from systemic physiological sources. The systemic physiology signals measured with SPA-fNIRS can be used for regressing out physiological confounding components in fNIRS signals. Misinterpretations can thus be minimized. (ii) SPA-fNIRS enables to study the embodied brain by linking the brain with the physiological state of the entire body, allowing novel insights into their complex interplay. We envisage the SPA-fNIRS approach will become increasingly important in the future.

## Highlights

• Systemic physiology augmented functional near-infrared spectroscopy (SPA-fNIRS)
• SPA-fNIRS enables an improved understanding of fNIRS signals
• SPA-fNIRS enables to avoid erroneous interpretations of fNIRS signals
• SPA-fNIRS enables to study the embodied brain

## 1 Neuroimaging with fNIRS: How to Correctly Interpret the Signals

### 1.1 fNIRS: Hemodynamic and Oxygenation in the Human Head Measured with Light

**Fig. 1:**
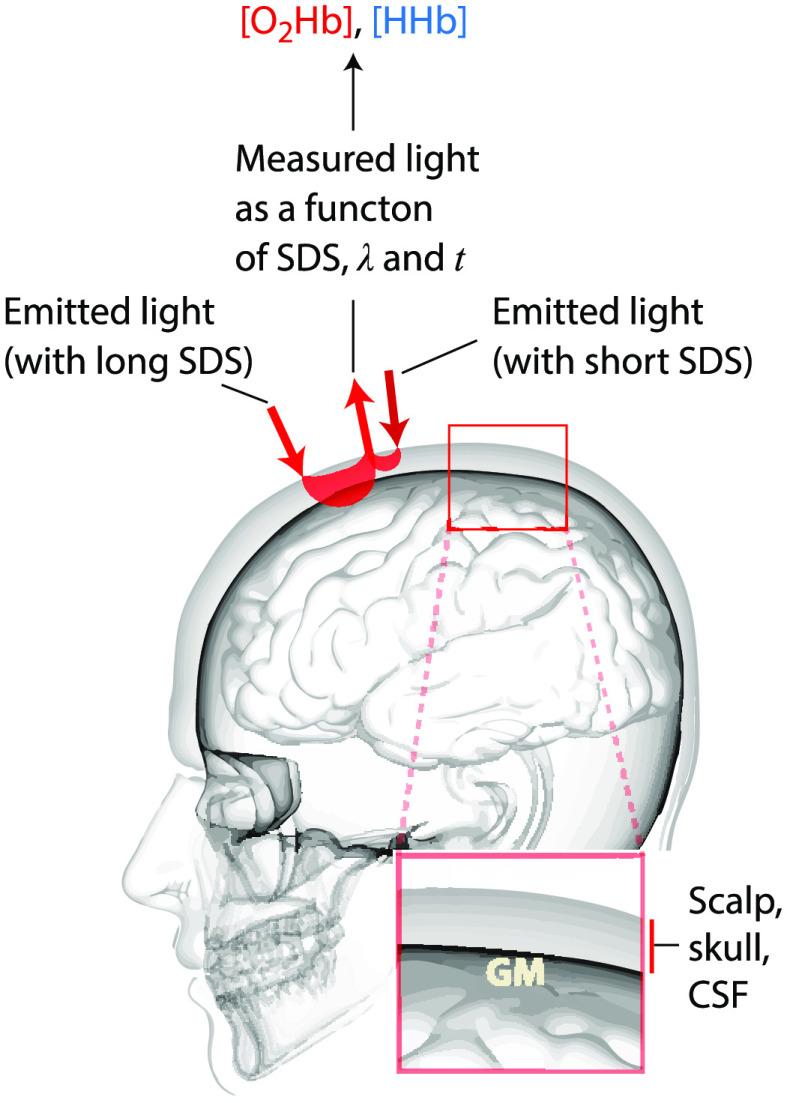
Basic principle of fNIRS measurements. Basic setup to record changes in cerebral hemodynamics and oxygenation on the human head with fNIRS. Light is shone into the tissue and the back-scattered light measured with a detector. A long and a short source-detector separation (SDS), forming long and short light channels, enable to distinguish between light absorption changes in the cerebral and extracerebral tissue. At least two wavelengths are used in the near-infrared range to separate the oxygenated ($\left[O_{2}\mathrm{Hb}\right]$) and deoxygenated ([HHb]) form of hemoglobin.

Optical neuroimaging in humans with functional near-infrared spectroscopy (fNIRS) is rapidly gaining popularity in neuroscience with an exponential increase of published papers over the last decades.^1^^,^^2^ Similar to functional magnetic resonance imaging (fMRI), fNIRS is a functional brain imaging technique based on detecting changes in hemodynamics and tissue oxygenation induced by neuronal activity [neurovascular coupling (NVC)]. fNIRS shines near-infrared (NIR) light (with at least two different wavelengths) in the head by placing light emitters on the scalp, and detecting the back-scattered light at specific distances apart. From this light intensity, the region and depth-dependent changes in the concentration of oxyhemoglobin ($\left[O_{2}\mathrm{Hb}\right]$), deoxyhemoglobin ([HHb]), and total hemoglobin ($\left[\mathrm{tHb}]=[O_{2}\mathrm{Hb}]+[\mathrm{HHb}\right]$) are calculated (Fig. 1).^3^

Over the past years, fNIRS was proven to be a valuable technique to study brain activity in humans of all ages, from preterm-born neonates (e.g., Karen et al.^4^) to elderly,^5^^,^^6^ in freely moving subjects in naturalistic environments,^7^ in several subjects in parallel (hyperscanning),^8^^,^^9^ as part of computer interfaces,^10^ and in combination with electroencephalography (EEG),^11^ fMRI,^12^^,^^13^ magnetoencephalography (MEG) (e.g., Huppert et al.^14^), positron emission tomography (PET) (e.g., Rostrup et al.^15^), or transcranial direct current stimulation (tDCS) (e.g., Di Rosa et al.^16^). Applications range from cognitive neuroscience experiments with healthy humans and patients with various disorders^2^^,^^17^ to more innovative applications such as the investigation of cerebral hemodynamic changes due to climbing,^18^ flying an aircraft,^19^ changes in sleep states,^20^^,^^21^ or due to the administration of psychedelics.^22^ fNIRS is an established neuroimaging technique with rapidly increasing popularity.

### 1.2 Complexity of Properly Interpreting fNIRS Signals

**Fig. 2:**
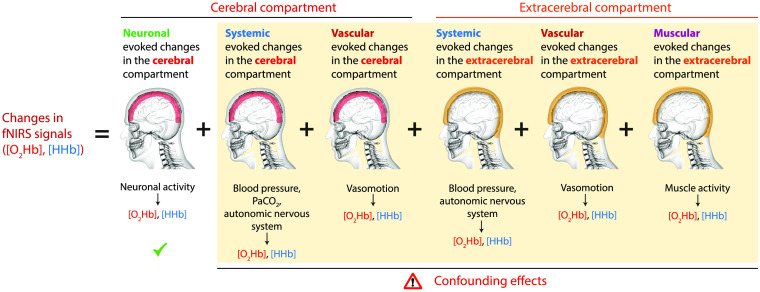
The components of the fNIRS signals. The six main components of the fNIRS signals, comprising three originating in the cerebral compartment and three in the extracerebral one. To investigate brain activity, only the first component is relevant and the other five are confounding the measurement. For a correct interpretation of the fNIRS signals, the components need to be discriminated and well characterized. The extracerebral component can be assessed by the short SDS, but the systemic effects need to be measured by separate methods.

Both fNIRS and fMRI are techniques that measure brain activity indirectly by determining the changes in hemodynamics and oxygenation elicited by NVC. But brain activity is not the only factor changing the hemodynamic signals measured with fMRI (blood-oxygen-level-dependent signal, BOLD, which reflects only the [HHb]) or fNIRS ($\left[O_{2}\mathrm{Hb}\right]$, [HHb], [tHb]). For fNIRS, all signal changes can be classified in a first approximation into three components: (i) neuronal evoked changes in the cerebral compartment (i.e., the component of interest when investigating brain activity), (ii) systemic evoked changes in the cerebral compartment, and (iii) systemic evoked changes in the extrecerebral compartment (for a detailed review, see Scholkmann et al.^3^ and Tachtsidis and Scholkmann^23^). Three additional components also play a role: (i) vascular evoked changes in the cerebral compartment, (ii) vascular evoked changes in the extracerebral compartment, and (iii) muscular evoked changes in the extracerebral compartment. The vascular evoked changes are due to vasomotion, i.e., spontaneous fluctuation in tone of blood vessel walls.^24^ The muscular evoked changes are induced by the activity of the temporal muscle on the head.^25^^,^^26^ The fNIRS signal thus comprises six components (Fig. 2). The composition of the fNIRS signal of these components is true for both evoked as well as spontaneous changes during the resting-state, and the individual contribution of each factor to the final measured signal varies. For the case of fMRI, the significance of non-neuronal drivers of the BOLD signal (e.g., systemic and vascular ones) is also increasingly recognized.^27^^,^^28^

Each fNIRS signal consists of these six components (Fig. 2). How much each component contributes to the overall fNIRS signal cannot be stated in general terms, as the weighting of the components depends on many factors, such as the experimental paradigm, measurement location, the technical implementation of the fNIRS measurement (e.g., measurement type and source–detector separation, SDS), as well as the individual physiology and anatomy of the subject. This highlights the need to measure correlates of these components with additional physiological techniques to obtain an overall assessment, which component is present and to what extent in the respective fNIRS measurement.

Two components not related to neuronal activity originate from changes in systemic physiological activity during the functional experiments, the main factors being the cardiorespiratory system (cardiac activity and respiration), the concentration of arterial partial-pressure of ${\mathrm{CO}}_{2}$ (${\mathrm{PaCO}}_{2}$), and the state of the autonomic nervous system (ANS).^23^

The non-neuronally driven physiological changes are a challenge for fNIRS (and likewise for fMRI) for two reasons: they may mimic a hemodynamic response normally observed due to an increase (or decrease) of brain activity (a “false positive”), or they may mask a neuronal induced hemodynamic response so that it is not detected (a “false negative”). For example, an increase in ${\mathrm{PaCO}}_{2}$ during the task can mimick a normal hemodynamic response, i.e., a large increase in $\left[O_{2}\mathrm{Hb}\right]$ and slight decrease in [HHb] (see Fig. 2 in Rostrup et al.^15^ and Fig. 2 in Amyot et al.^29^).

**Fig. 3:**
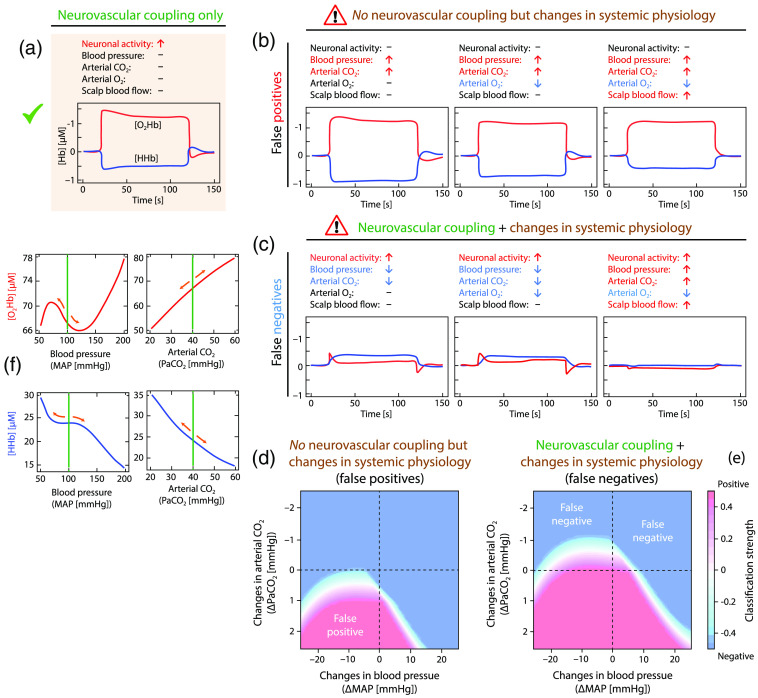
How changes in systemic physiological activity interfere with fNIRS signals. (a) Typical changes in fNIRS signals due to NVC (i.e., increase in $\left[O_{2}\mathrm{Hb}\right]$ and decrease in [HHb]). (b) The same pattern as in (a) can be obtained without increase in brain activity, but solely by changes in systemic physiology that mimic the typical NVC pattern. False-positive detections of brain activity are the result. (c) When both brain activity and systemic physiological activity change, the cerebrovascular regulatory processes may destructively interfere and leaving the fNIRS signal unchanged, leading to false-negative results. (d) and (e) visualize the probabilities of the occurrences of false-positives and negatives as a function of changes in ${\mathrm{PaCO}}_{2}$ and blood pressure in the case of no change in brain activity (d) and an increase in brain activity (e). The relationship between changes in fNIRS signals and changes in systemic physiological signals are highly non-linear, as exemplarily shown in (f). The results presented in (a)–(f) were obtained by a computational physiological model investigated by us^30^ and based on the assumption of a baseline MAP of 100 mm Hg and a ${\mathrm{PaCO}}_{2}$ of 40 mm Hg. Shifting the baseline ${\mathrm{PaCO}}_{2}$ and MAP values along the curves shown in (f) will result in other results than shown in (b)–(e) highlighting the complex interplay between the cerebrovascular regulation mechanisms. The small orange arrows in (f) indicate the direction the fNIRS signals can change when changes in ${\mathrm{PaCO}}_{2}$ and MAP occur with respect to the standard baseline values. Note the strong non-linear relationship between $\left[O_{2}\mathrm{Hb}\right]$ and MAP: both are positively related in the MAP range of about 50 to 70 mm Hg, negatively in the range of about 75 to 125 mm Hg, and positively above ∼125  mm Hg.

A detailed study of how changes in systemic physiological activity lead to characteristic changes in the fNIRS signals has been performed by us with computational modeling of the systemic and cerebral physiology. The results highlight the importance of being aware of this context when performing fNIRS studies.^30^ Figure 3 shows the results of our investigation.

On one hand, it is surprising that the large impact of such systemic physiological changes on fNIRS and fMRI signals has been rather ignored until recently. On the other hand, this is understandable for the following reason: traditionally, it was thought that possible changes in systemic physiology during a functional neuroimaging experiment are “damped” and diminished in the cerebral (and extracerebral) compartment of the head and consequentely not interfering with detecting the brain activity related fNIRS signal component (experience from personal conversations). The reason for this attitude is most likely the prevailing oversimplified view of cerebral blood flow (CBF) and oxygenation being maintained at a constant level. However, the understanding of the two classical mechanisms responsible for that, i.e., cerebral autoregulation (CA) and cerebrovascular reactivity (CVR), changed significantly over the last decades (for a detailed summary of the history of the understanding of CA and CVR, see the next section). In addition, some recent studies show the significance of the ANS in CVR, i.e., influencing cerebral and extracerebral blood flow.^31^ Furthermore, the different cerebrovascular regulation mechanisms (i.e., NVC, CA, CVR, and ANS-related regulation) are interacting, rendering oversimplified physiological assumptions obsolete.

Systemic physiological changes are also influencing BOLD-fMRI measurements.^27^^,^^28^^,^^32^ The discovery of resting-state “physiological networks” that are linked to well-characterized resting-state neuronal networks,^33^ or the insight that sympathetic activity contributes to the fMRI signal more than previously known,^31^ are recent examples of novel insights into this matter, which appear at an increasing rate.

From these insights, it can be inferred directly that there is a need to monitor changes in systemic physiology when performing fNIRS and fMRI studies to increase the chance of interpreting the signals correctly.

### 1.3 Cerebral Autoregulation: From an Oversimplified to a More Realistic Understanding

**Fig. 4:**
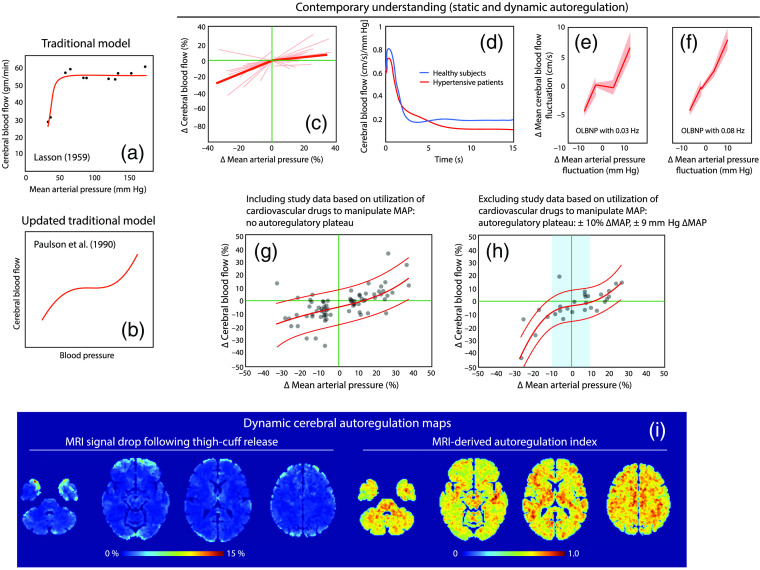
Visualization of key insights into cerebral autoregulation. While the (a) traditional model erroneously stated that CA enables to have a constant CBF during a wide range of MAP values,^36^ an (b) updated version acknowledged the non-linear function of the relationship^37^ The modern understanding is that the stable plateau, where CBF remains constant, is (c) quite narrow and the function describing the two parameters (CBF and MAP) in a static case depends also on the (c) individual subject (see the individual orange thin lines).^38^ (d) Changes in CBF due to a sharp step-increase in MAP.^39^ Dynamic CA is not able to suppress the initial sharp increase in CBF. (d)–(f) Examples of dynamic CAs for two different frequencies of oscillatory blood pressure changes induced by lower body negative pressure. Results from a study in humans.^40^ Note the different ability of dynamic CA to maintain constant CBF. (g) and (h) CA functions according to a recent meta-analysis of published studies.^34^ Shown are the relationships for (g) including and (h) excluding studies that used cardiovascular drugs to induce changes in MAP. A pressure-passive relationship is present for the (g) pharmacological data whereas the non-pharmacological data show a narrow plateau. (i) Dynamic CA maps obtained by fMRI with a gradient-echo echo-planar pulse sequence.^41^ Dynamic CA was quantified by the MRI-signal drop magnitude following thigh-cuff release (images left) or by a MRI-derived autoregulation index based on the signal recovery function (images right). Both parameters showed no regional dependence (frontal, occipital, parietal, and temporal cortices) but gray matter was showing a greater signal drop and a slower recovery compared to white matter. There was a tendency of a larger drop in the prefrontal and occipital cortices (albeit not statistically significant due to the large intra-subject variability). OLBNP: oscillatory lower body negative pressure. (a)–(h) Redrawn from respective articles. (i) Modified from Horsfield et al.^41^

**Fig. 5:**
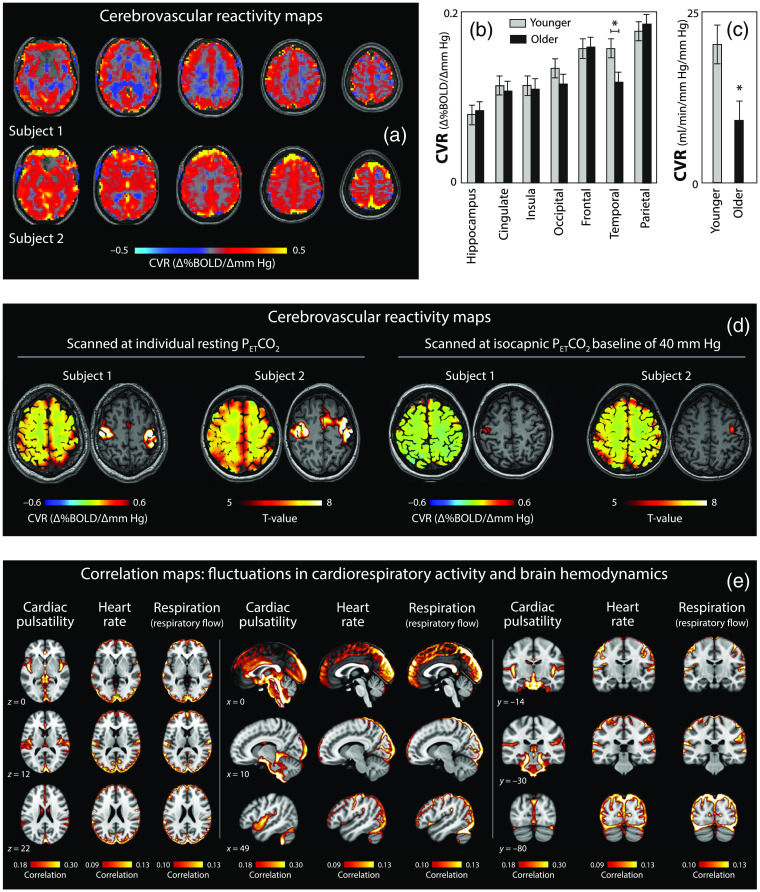
The region-dependency of CVR and impact of fluctuations in cardiorespiratory activity on brain hemodynamics. (a) CVR maps of two healthy subjects.^46^ Note the differences of the maps for the two subject, highlighting the significance of inter-subject variability of CVR. In both cases, the CVR is higher in cortical regions compared to interior parts of the brain. Subject 2 has a more pronounced CVR in the frontal and occipital lobes compared to the temporal regions. (b) Spatial heterogeneity of CVR and the influence of age.^47^ According to this study, the parietal lobe shows the strongest CVR, followed by the temporal, frontal and occipital lobes. Aging was shown to decrease the CVR in the temporal lobe in particular. (c) Age-related decline in CVR.^48^ Shown is the drop in CVR caused by aging (CVR averaged over the whole brain; 25±3 years versus 61±5 years). (d) The CVR and task-evoke hemodynamic changes depend on the individual resting $P_{\mathrm{ET}}{\mathrm{CO}}_{2}$ level.^49^ Examples from two subjects. fMRI scanning was performed two times per subject; one time at individual resting $P_{\mathrm{ET}}{\mathrm{CO}}_{2}$, the other at an isocapnic $P_{\mathrm{ET}}{\mathrm{CO}}_{2}$ baseline at 40 mm Hg. Scanning at 40 mm Hg $P_{\mathrm{ET}}{\mathrm{CO}}_{2}$ causes a reduction in CVR as well as a reduction in the amplitude of hemodynamic changes evoked by bilateral finger-tapping. This study highlights the importance how the baseline $P_{\mathrm{ET}}{\mathrm{CO}}_{2}$ level is influencing neuroimaging results. (e) Correlation maps showing region-dependent influences of cardiorespiratory activity on brain hemodynamics.^45^ The correlations with cardiac pulsatility was determined with the RETROICOR method, HR and respiration influences with a scan-specific models. Note the large correlations of HR and respiration with hemodynamic fluctuations in the frontal and occipital lobes. Subfigures (a), (d) and (e) modified from Williams et al.,^46^ van Niftrik et al.,^49^ and Kassinopoulos and Mitsis.^45^ Subfigures (b) and (c) redrawn from Catchlove et al.^47^ and Miller et al.^48^

The understanding of CA changed quite significantly over the last decades.^34^^,^^35^ Introduced by a review of Lassen,^36^ CA was regarded as maintaining CBF over a large mean arterial blood pressure (MAP) range (50 to 150 mm Hg) [Fig. 4(a)]. This model was updated decades later by introduction of an upper limit of the autoregulatory curve^37^ [Fig. 4(b)]. The modern understanding of CA is, however, that (i) the original conclusion of Lassen is basically an artefact due to wrong data analysis and interpretation,^34^^,^^35^ (ii) the stable plateau of CBF is much more narrow and inter-individual variations of CA are large [Figs. 3(c) and 3(h)], sharp transients in MAP are only marginally damped by CA [Fig. 3(d)], and static and dynamic CA need to be distinguished: while static CA reflects the change in CBF to a steady-state change in MAP, dynamic CA describes the frequency-dependent CA ability in response to MAP fluctuations [Figs. 3(e) and 3(f)]. The presence and range of the autoregulatory plateau depends also how the data is analyzed (taking into account study results that utilized cardiovascular drugs to manipulate MAP or not)^34^ [Figs. 4(g) and 4(h)]. Furthermore, according to the new understanding of CA, the strength and characteristics of CA are influenced by other cerebrovascular regulation mechanisms such as NVC and CVR as well as baseline physiology (e.g., end-tidal ${\mathrm{CO}}_{2}$, i.e., $P_{\mathrm{ET}}{\mathrm{CO}}_{2}$^42^). Evidence is also accumulating that not only the arterial but also the venous part of the vasculature performs CA.^43^ In addition, there is an asymmetry of dynamic CA (weaker autoregulation with an increase in blood pressure compared to a decrease)—something for which there is growing evidence but which is usually not taken into account by the commonly used methods for CA determination since they assume autoregulatory responses to be symmetric.^44^ With regard to a possible region-dependence of CA, gray matter has a greater drop and slower recovery of CBF induced by a change in MAP compared to white matter, and the prefrontal and occipital lobe showed a tendency to have a difference CA compared to the other regions of the cerebral cortex^41^ [Fig. 4(i)]. Changes in cardiorespiratory activity have a global impact on brain hemodynamics with stronger impacts on hemodynamics in the frontal and occipital lobes^45^ [Fig. 5(e)]. The cardiorespiratory impact on cerebral hemodynamics is thus quite heterogeneous.

All these modern insights into CA are very relevant for fNIRS since they show that CBF and thus $\left[O_{2}\mathrm{Hb},\right]$ [HHb], and [tHb] are not as independent of MAP as previously thought, explaining how changes in systemic physiology can have a significant impact on the fNIRS signals.

### 1.4 Cerebrovascular Reactivity: The Complex Impact of Changes in PaCO_2_ on Cerebral Hemodynamics

CVR is the vascular response to a vasoactive agent such as ${\mathrm{PaCO}}_{2}$. The strength of CVR in the human brain vasculature is determined by many factors, including age [Figs. 5(b) and 5(c)],^47^^,^^48^ the baseline resting ${\mathrm{PaCO}}_{2}$ level [Fig. 5(d)],^49^ the individual subject [Fig. 5(a)]^46^ and sex.^50^ The resting-state individual baseline $P_{a}{\mathrm{CO}}_{2}$ determines the individual CVR as well as task-evoked hemodynamic responses^49^ [Fig. 5(d)]. These aspects of CVR need to be considered when performing functional neuroimaging studies with fNIRS and fMRI to enable a correct interpretation of the hemodynamic signals measured. Furthermore, ${\mathrm{PaCO}}_{2}$ often changes even during simple and easy tasks,^8^ which may lead to false-positive or false-negative responses^30^ and thus to misinterpretations of the data.

### 1.5 Need to Fully Appreciate How Human Brain Activity and Cerebral Blood Flow Regulation is Linked to Systemic Body Physiology

Knowing how much systemic physiology is changing during a functional task not only assists to remove these confounding effects, it also enables to determine how systemic physiology is modulating NVC as well as neuronal brain activity: Monitoring systemic physiological changes thus enables insights into body-brain interactions. This is of high importance for neuroimaging studies.

#### 1.5.1 Systemic physiology modulates neurovascular coupling and brain activity

Systemic physiology has an impact on NVC. ${\mathrm{PaCO}}_{2}$ is particularly relevant in this case due to its strong vasomodulatory effect. For example, an increase in ${\mathrm{PaCO}}_{2}$ (hypercapnia) is causing vasodilation, resulting in a decreased vasodilatatory capacity, and thus, a damped NVC,^49^ whereas a decrease in ${\mathrm{PaCO}}_{2}$ (hypocapnia) induces vasoconstriction and thus interferes with NVC too.^8^^,^^51^^,^^52^

Not only NVC induced by a stimulus or a task may be affected, but also the NVC underlying resting-state functional connectivity strength is modulated by ${\mathrm{PaCO}}_{2}$, i.e., hypocapnia leads to the highest connectivity, hypercapnia leads to the lowest connectivity^53^ and it is well known that resting-state cerebral hemodynamics fluctuates in synchrony with spontaneous changes in ${\mathrm{PaCO}}_{2}$.^54^^,^^55^ Also, as already mentioned, the baseline ${\mathrm{PaCO}}_{2}$ can determine the magnitude of task-evoked hemodynamic responses.^49^

Specific diseases are also associated with alterations, in general a reduction of NVC, e.g., type 2 diabetes,^56^ hypertension,^57^ chronic migraine,^58^ Alzheimer’s disease,^59^ cerebro-occlusive disease,^60^ or atrial fibrillation.^61^ Alternations in NVC (increases and decreases, depending on study) were also found in multiple sclerosis.^62^^,^^63^ Vascular oxidative stress seems to be a common factor involved in these diseases linked to cerebrovascular dysfunction and subsequent alternations in NVC.^64^

The cardiorespiratory fitness of the subject also affects the NVC and thus the amplitude of the cerebral hemodynamic response^65^^,^^66^

The fact that systemic physiology modulates NVC is not new: It has already been pointed out 10 years ago by Lindauer et al.^67^ that interpreting hemodynamic signals as measured with fMRI and fNIRS as solely representing brain activity neglects the relevant influence of the physiological situation and thus leads to erroneous conclusions.^30^

Conversely, brain activity is also directly influenced by systemic physiological activity. Again here, ${\mathrm{PaCO}}_{2}$, is an important parameter to consider. For example, induced hypercapnia causes a decrease in the power of brain oscillations^68^^–^^70^ and a reduction in the cerebral metabolic rate of oxygen.^69^ Even very mild levels of hypercapnia in humans cause a reduction in alpha-, beta-, and gamma-band power, and even the small spontaneous changes in ${\mathrm{PaCO}}_{2}$ that occur during normal breathing were found to influence neuronal oscillatory power significantly.^70^ Involuntarily changes in breathing (frequency and depth) during task performance may have an effect on neuronal oscillatory power.^70^ Changes in tidal-volume and respiration phase are also found to affect brain activity directly.^71^ Recent work pointed out that respiration-entrained brain rhythms are significant but unfortunately “often overlooked” in studies^72^ and that there is a tight interaction between respiration, brain activity, and cognition.^73^

In addition to ${\mathrm{PaCO}}_{2}$ and respiration, cardiac activity also has a direct effect on electrical brain activity. For example, heartbeats evoke cortical responses (so-called “heartbeat-evoked responses”) lead to an increase in specific cortical regions (the “heartbeat-induced network”), which is linked for example to the person’s mood^74^ and plays a role in somatosensory perception.^75^ The heart activity itself also directly influences conscious perception.^75^

Furthermore, even the stomach has a direct effect on brain activity: spontaneous alpha rhythm fluctuations in the brain show a phase-amplitude coupling with gastric activity,^76^ and a specific resting-state network is associated with the stomach–brain interaction.^77^ Moreover, there are complex central nervous system–peripheral nervous system relationships with the heart activity playing an important role (as outlined by the neurovisceral integration model by Thayer et al.^78^^,^^79^).

Finally, baseline blood pressure of a subject during the resting-phase before the start of the experiment (task or stimulation) has been recently shown by our group to be correlated with the individual difference of hemodynamic changes at the visual cortex (VC) between two colored light exposures (red and blue).^80^

In conclusion, there is ample evidence that brain activity is modulated by systemic physiology.

#### 1.5.2 Towards a truly embedded neuroscience: appreciating body-brain interactions

The section above underlines that it is important to measure systemic physiological signals during fNIRS neuroimaging experiments: Systemic physiology not only interferes with the measured signals (making the interpretation difficult) but also NVC and brain activity are directly modulated by systemic physiology. Different modular bi-directional interactions between the brain and the body (e.g., respiration-brain and heart-brain coupling) need to be included in the study of the brain to see the whole picture. Consequently, focusing only on the brain and the mind when doing experiments in the field of cognitive neuroscience is not sufficient. For several decades, it has been clear that the brain is embodied and it is advisable to study it accordingly.^81^ An embodied cognitive neuroscience is needed to study the dynamical interaction between the brain and the body embedded in the person’s environment.^82^

## 2 Systemic Physiology Augmented Functional Near-Infrared Spectroscopy

### 2.1 Solution for a Deeper Understanding of fNIRS Signals

**Fig. 6:**
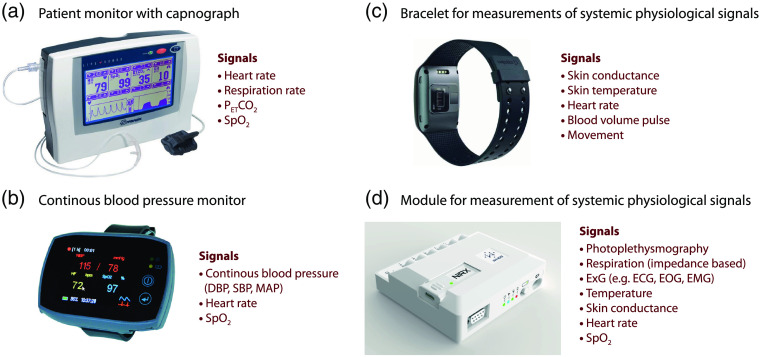
Examples of devices to measures systemic physiological signals. (a) Patient monitor LifeSense, Nonin Medical Inc., Plymouth, MN, USA. (b) Device for continuous noninvasive blood pressure monitoring; SOMNOtouch NIBP, SOMNOmedics, Germany. (c) E4 bracelet from Empatica Inc., Cambridge, MA, USA. (d) NIRxWINGS, NIRx, NIRx Medizintechnik GmbH, Berlin, Germany. The device is a module that can be linked to the NIRSport2 fNIRS device. (a)–(d) Adopted with permission from nonin.com, somnomedics.de, and empatica.com, and nirx.net, respectively.

**Fig. 7:**
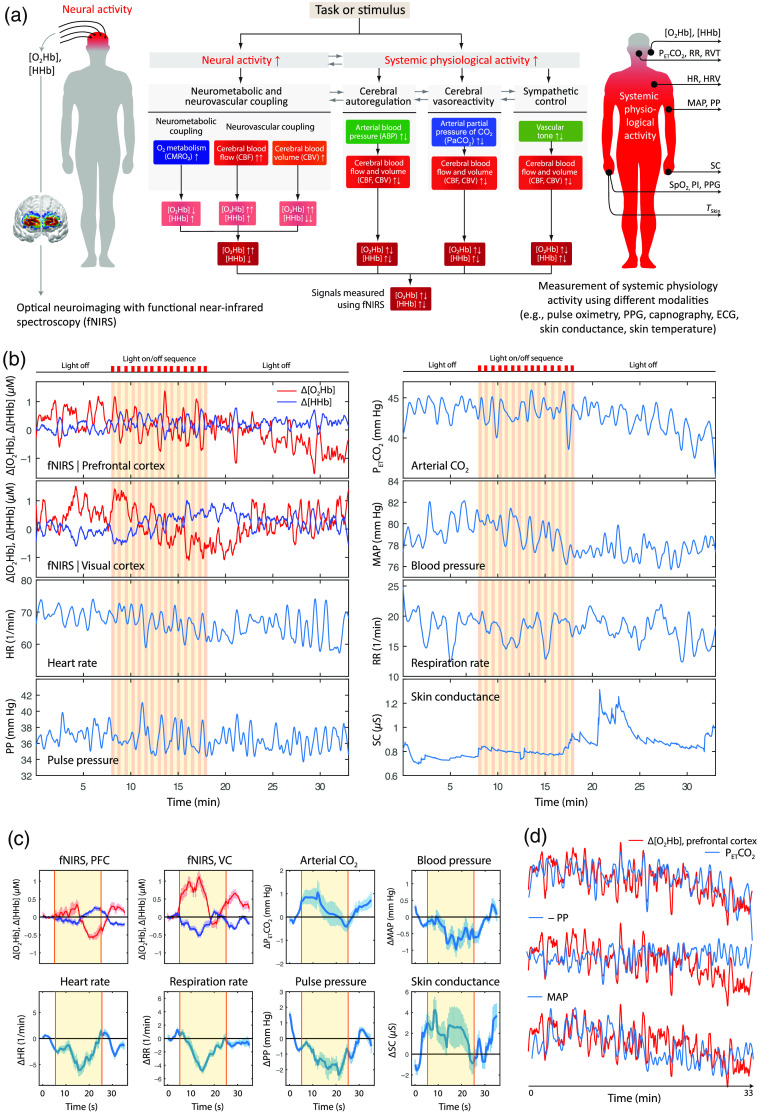
SPA-fNIRS: Schematic and application example. (a) Visualization of the SPA-fNIRS approach that simultaneously measures brain activity with fNIRS and systemic physiological activity with additional devices. The four main cerebrovascular regulation mechanisms are depicted that mediate task or stimulus evoked changes in the fNIRS signals. (b) Example of changes in fNIRS signals measured at the prefrontal cortex (PFC) and visual cortex (VC) as well as systemic physiological signals exemplarily from one subject during an experiment with a visual stimulation (20 s light on/off sequence for 10 min). (c) Block average of fNIRS and systemic physiological signals. It can be clearly seen that the visual stimulation elicits a characteristic hemodynamic response at the VC and that there are also stimulus-evoked changes in the systemic physiological signals. (d) When comparing the long-term trend of the fNIRS signals the trend in the fNIRS signals (in this case $\left[O_{2}\mathrm{Hb}\right]$ at the PFC) correlates well with the trend in systemic physiological signals (e.g., $P_{\mathrm{ET}}{\mathrm{CO}}_{2}$, PP, and MAP). Note that the correlation (strength and sign) is frequency and signal dependent. For example, the long-term trend in $\left[O_{2}\mathrm{Hb}\right]$ correlates strongly with $P_{\mathrm{ET}}{\mathrm{CO}}_{2}$ (Pearson’s r=0.711 (95% CI: [0.679, 0.741]), p<0.001) and MAP (r=0.520 (95% CI: [0.473, 0.564]), p<0,001) whereas the high-frequency part of PP is negatively correlated (correlation between linearly detrended time-series: r=−0.305 (95% CI: [−0.369,−0.248], p<0.01). Unpublished data from own measurements. $\left[O_{2}\mathrm{Hb}\right]$: concentration of oxyhemoglobin, [HHb]: concentration of deoxyhemoglobin, $P_{\mathrm{ET}}{\mathrm{CO}}_{2}$: end-tidal ${\mathrm{CO}}_{2}$, RR: respiration rate, RVT: respiration volume per time, HR: heart rate, HRV: heart rate variability, SC: skin conductance, ${\mathrm{SpO}}_{2}$: arterial oxygen saturation measured using pulse oximetry, MAP: mean arterial pressure, PP: pulse pressure, PI: perfusion index, PPG: photoplethysmogram, and $T_{\text{Skin}}$: skin temperature.

**Fig. 8:**
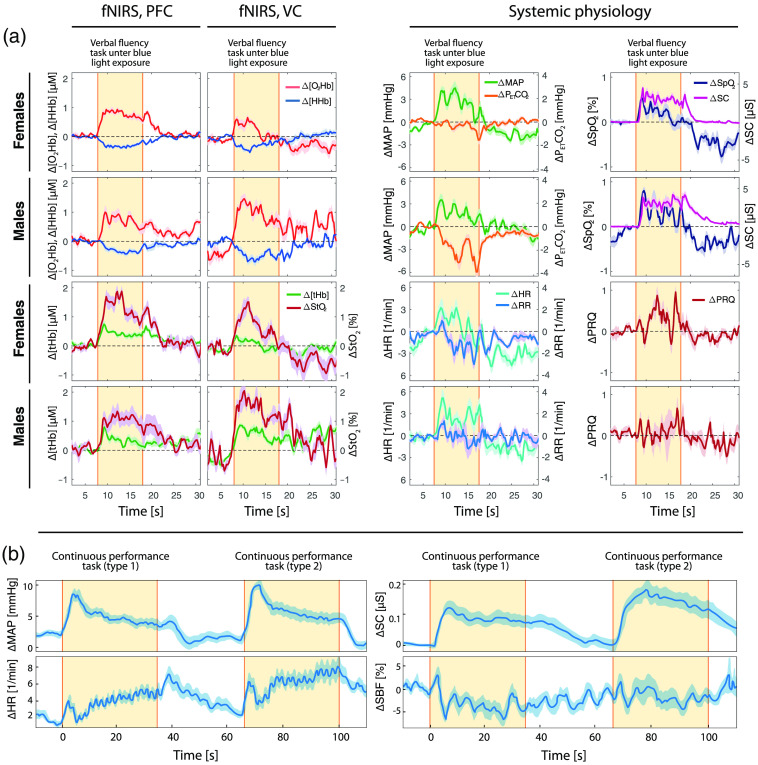
SPA-fNIRS application examples. (a) Changes in fNIRS signals measured on the PFC and VC in a SPA-fNIRS study investigating the effect of a verbal fluency task under a continuous long-term blue light exposure.^88^ The increases in $\left[O_{2}\mathrm{Hb}\right]$, ${\mathrm{StO}}_{2}$, and [tHb] at the PFC and VC are clearly accompanied by increases in MAP, HR, ${\mathrm{SpO}}_{2}$, and SC. It is worth noting the large drop in $P_{\mathrm{ET}}{\mathrm{CO}}_{2}$ in males and a similarity of the fluctuations of $\left[O_{2}\mathrm{Hb}\right]$ from the PFC and $P_{\mathrm{ET}}{\mathrm{CO}}_{2}$ as well as RR in males. (b) Changes in MAP, HR, SC, and scalp blood flow (SBF) recorded in a SPA-fNIRS study involving a continuous performance task (involving two task variants).^89^ Strong increases in MAP and SC can be seen at the beginning of each task epoch. (a) Redrawn from Zohdi et al.^88^ and (b) redrawn from Kirilina et al.^89^

In 2013, our research groups (Bern and Zurich) realized that changes in ${\mathrm{PaCO}}_{2}$ could play a major role in fNIRS studies whenever changes in breathing are expected to take place.^8^ In the following, we found that mild stimuli unexpectedly lead to changes in ${\mathrm{PaCO}}_{2}$.^83^^,^^84^ In a further study, we also observed significant stimulus-triggered drops in ${\mathrm{PaCO}}_{2}$ during repeated mechanical pain stimulation at the lower back that interfered with the fNIRS signals measured at the head.^85^ Subsequently, we developed a new measurement setup that enables the measurement of fNIRS signals from the brain and systemic physiological signals from the body simultaneously (see Fig. 6 for examples of such a measurement setup), and a new term was introduced in 2017 to refer to this measurement approach: “systemic physiology augmented functional near-infrared spectroscopy” (SPA-fNIRS).^86^^,^^87^ Figures 7 and 8 show how SPA-fNIRS works and provides example of signals measured during a functional tasks.

When employing the SPA-fNIRS approach, two main questions need to be answered: which systemic physiologic signals should be recorded? And how should they be analyzed?

We recommend to record at least the physiological signals that cover the activity of the cardio-respiratory system and the ANS, i.e., the ${\mathrm{PaCO}}_{2}$, blood pressure, respiration rate (RR), heart rate (HR), arterial oxygen saturation, and skin conductance (SC). This can be achieved non-invasively with respective devices (see Fig. 6), and it is safer to include these measurements since even mild stimuli may evoke unexpected physiological changes, particularly in the ${\mathrm{PaCO}}_{2}$. An additional interesting signal may be photoplethysmography (PPG) measured at the periphery (finger, ear, and toe).^90^^–^^92^ Assessing the changes in the state of the ANS is also important due to the impact of the ANS on cerebral hemodynamics^31^^,^^93^—an aspect generally underappreciated in fMRI and fNIRS studies. Also, the investigation of correlations between brain activity and ANS activity, as directly assessed by SC, is relevant.^94^ Although some of the variables are connected, e.g., the breathing rate to the ${\mathrm{PaCO}}_{2}$, it is recommended to measure both, because the quantitative size in the change of ${\mathrm{PaCO}}_{2}$ cannot be inferred from the change in the breathing rate. In addition, all these parameters are measured non-invasively and are, therefore, not a burden for the subject. Concerning the question how the systemic physiological signals should be processed and analyzed within the SPA-fNIRS approach, there is currently no standard procedure available, and the research how to analyze fNIRS data along systemic physiological data just started. Different methods have been employed so far, including the calculation of block-averages of stimulus-evoked changes in the signals,^95^^,^^96^ block-averaging with subsequent correlation analysis to investigate the neurosystemic functional connectivity,^87^ the use of a general linear model (GLM) that treats the systemic physiological signals as additional regressors,^89^^,^^91^^,^^92^^,^^97^^–^^99^ wavelet coherence analysis,^97^ the use coupling functions derived from the phases of the signals via the continuous wavelet transform,^24^ oblique subspace projections signal decomposition,^100^ or the recent approach using a GLM and regularized temporally embedded canonical correlation analysis (tCCA).^27^^,^^101^ The use of tCCA allows one to create optimal nuisance regressors by considering non-instantaneous and non-constant coupling between the recorded signals and by intelligently combining any available auxiliary signals (e.g., systemic physiology and short-channel fNIRS recordings). Combining tCCA with GLM leads generally to improved detection of task-or stimulus-evoked hemodynamic responses in case of the presence of systemic physiological confounders, a low contrast-to-noise-ratio of the fNIRS signals and a low number of stimuli or trials.^101^

### 2.2 Solution to Study the Embodied Human Brain

SPA-fNIRS provides a new opportunity to study body–brain interactions. For example, in an SPA-fNIRS study, we investigated functional physiological changes due to visual stimulation (20 s light on/off sequence) we observed large changes in fNIRS and systemic physiological signals with a significant inter-individual variability especially evident in the systemic physiological changes.^87^ Stimulus-evoked changes in all systemic physiological signals were observed. Interestingly, the time-course of fNIRS signals showed correlations with changes in the heart rate variability (HRV) as well as the power of spontaneous low-frequency oscillations (Mayer waves), indicating that the cardiac system and ANS were modulated by the stimulation. We showed that a significant amount of variability in the fNIRS signals was caused by changes in systemic physiology. If no systemic physiological signals were recorded, erroneous conclusions would have been drawn (i.e., the changes in the fNIRS signals would have been interpreted as caused by NVC alone). In addition, the intriguing coordination and interaction between body and brain would have been missed. In our newest SPA-fNIRS study, we replicated the findings of the significance of evoked systemic physiological in experimental paradigm with visual stimulations and showed the importance to perform a single-subject analysis to classify the pattern in fNIRS and systemic physiological signals into different groups.^95^ In this study, we found that shapes of the hemodynamic responses in the fNIRS signals induced by a long-term visual stimulation can be classified into different groups, and that these groups related to specific changes in systemic physiology. Pioneering work has been also done by others, including the group of de Frederick who showed that changes in tissue oxygenation/hemodynamics measured with a NIRS probe on the finger and toe are highly correlated with the BOLD fMRI signal; the peripheral NIRS signal was found to be strongly correlated to some functional resting-state networks.^102^

### 2.3 Useful Devices for Extending fNIRS to SPA-fNIRS Measurements

Nowadays, the measurement of changes in systemic physiological signals can be performed with a minimal and affordable setup of non-invasive devices. Some basic instrumentation that we recommend may even be included in a patient monitor: a capnograph to measure $P_{\mathrm{ET}}{\mathrm{CO}}_{2}$, an electrocardiograph for HR and RR, and a pulse oximeter for ${\mathrm{SpO}}_{2}$ [Fig. 6(a)]. In addition, we recommend to include a device that enables measurement of the blood pressure continuously and non-invasively. There are a few devices on the market [Fig. 6(b)]. These additional physiological measurements are also perfectly feasible in freely moving subjects since wearable versions of the devices are available. A PPG and/or ${\mathrm{StO}}_{2}$ sensor is easily attached to an extremity of children or neonates for SPA-fNIRS, as we know from our own experience.

Worth mentioning is also an in-house developed multichannel NIRS oximeter that can measure pulse waves at different peripheral body positions (e.g., fingers, toes, and ear lobe), allowing investigation of the dynamic and causal relationships of brain versus systemic haemodynamic responses.^90^ For the measurement of SC, miniaturized high-precision devices are available that are for example worn on the wrist like a watch [Fig. 6(c)]. We recommend two devices, which enable the measurement on the left and the right hand in parallel, enabling novel ways to assess the state of the ANS.^28^ This device can also measure the body temperature. All of these devices need to be certified for the application, allow synchronization, and be able to record the data, obviously a prerequisite for SPA-fNIRS studies. A device especially designed for measurements of systemic physiology along fNIRS neuroimaging signals was introduced recently [Fig. 6(d)].

This collection of examples shows that the additional measurement of parameters of systemic physiology is simply, non-invasive and, as pointed out above, provides important data.

### 2.4 Questions and Answers

Below is a list of answers to frequently asked questions about SPA-fNIRS.

Question (Q): What is SPA-fNIRS?

Answer (A): SPA-fNIRS is a method that enables the measurement and analysis of fNIRS neuroimaging data along with data from systemic physiology.

Q: What is the benefit of using SPA-fNIRS instead of “traditional” fNIRS?

A: The fNIRS signals comprise different components (Fig. 2), including those related to changes in systemic physiology and not to NVC. SPA-fNIRS allows one to disentangle the fNIRS signal by incorporating the information of the measured changes in systemic physiology as well as to investigate the relationship between fNIRS signals and systemic physiology. This reduces the likelihood of misinterpretations of the fNIRS signal changes and enables a deeper understanding of the interplay between brain activity and systemic physiology.

Q: Which systemic physiological signals are recommended to be measured for a SPA-fNIRS study? What are the most important ones?

A: We recommend to measure primarily systemic physiological signals related to the state of the cardiorespiratory sytem and ANS, i.e., HR, RR, MAP, $P_{\mathrm{ET}}{\mathrm{CO}}_{2}$, ${\mathrm{SpO}}_{2}$, and SC. MAP and $P_{\mathrm{ET}}{\mathrm{CO}}_{2}$ are highly relevant since they directly affect the cerebral blood flow. HR, RR, and SC are the easiest measurements to achieve and provide important information about the systemic physiological status of the subjects. ${\mathrm{SpO}}_{2}$ may be less relevant in healthy subjects but is easy to perform. We recommend to select the physiological signals based on the task or stimulus of the study. For example, when the stimulus is expected to change the breathing (e.g., due to speaking), $P_{\mathrm{ET}}{\mathrm{CO}}_{2}$ is recommended to be measured.^8^ If the experimental protocol is inducing stress in the subject, SC, HR, and MAP measurements are recommended.

Studies are ongoing whether the PPG signal measured peripherally can be used as a surrogate for several cardiorespiratory and ANS parameters. In addition, the information in the fNIRS short-channels includes valuable information about systemic physiological changes. How the information from PPG signals and from short-channels can be used in a SPA-fNIRS context to derive systemic physiological information from them is currently under investigation.

Q: Do changes in systemic physiology affect the hemodynamics on the head homogenously? Are there regional differences?

A: As shown in Figs. 4 and 5, CVR and CA are valid at first approximation for the whole brain, but regional differences are present, especially for CVR. The PFC, often measured in fNIRS studies, is affected by changes in ${\mathrm{PaCO}}_{2}$ (CVR), MAP (CA) and ANS. The regional-differences of changes in systemic physiology are important to be considered when aiming for a precise and error-free interpretation of fNIRS data since the assumption that the whole brain is similarly affected by the systemic physiology is only true in the first approximation. Therefore, the notion that all regional-differences can be attributed only to NVC is not quite correct.

Q: How should the SPA-fNIRS data be processed and analyzed?

A: The optimal use of systemic physiological data along with fNIRS data is currently further refined. The physiological data enables for example to regress out (e.g., using a GLM) the influence of changes in systemic physiology on fNIRS signals or to investigate the interplay between the brain and body (i.e., neurosystemic functional connectivity). We expect a large increase in the refinement of signal-processing and data analysis methods and frameworks for SPA-fNIRS data in the near future. With regard to the use of SPA-fNIRS in combination with GLM data analysis, multicollinearity of the covariates could be a problem.^103^ Multicolinearity is an issue to be aware of with any regression analysis. The presence of multicollinearity can be assessed for example by the variance inflation factor^104^ or the weighted variance inflation factor,^105^ and the problem can be minimized by combining correlated variables to a single variable or by employing regression algorithms that can deal with multicollinearity (e.g., Ridge regression and Lasso regression).^106^ In addition, it is necessary to carefully design the GLM regression matrix given the SPA-fNIRS data to avoid underfitting and overfitting. Cross-validated Bayesian model selection is a solution for this issue.^107^ For all SPA-fNIRS signal-processing, it might also be important to not use the systemic physiological signals in the regression models directly but to perform a convolution of the signals with respective “physiological response functions” (PRFs) first. Such PRFs have been already determined for fMRI^45^^,^^108^^,^^109^ and are expected to be useful and necessary for SPA-fNIRS data processing too. Since the PRFs vary between subjects,^45^ the PRFs should be determined ideally for each subject (and experiment) separately. It is expected that the use of individual PRFs will improve the GLM regression of SPA-fNIRS data. Unfortunately, generally valid PRFs for SPA-fNIRS data have not been published so far and ways to determine subject-specific or session-specific PRFs still need to be developed. Innovative research contributions are encouraged in this area. Alternatively, the time-dependent wavelet coherence in specific frequency band between fNIRS signals and systemic physiological signals can be used as a regressor in a GLM framework, as first shown by Kirilina et al.^97^. Furthermore, general PRFs might be also derived by physiological modeling.^30^^,^^110^^–^^112^

Q: Are the software available to perform the SPA-fNIRS data processing and analysis?

A: There currently is no software available yet specifically designed for SPA-fNIRS data processing and analysis. However, currently available software, e.g., Homer3 (https://github.com/BUNPC/Homer3),^113^ NIRS-SPM,^114^ NIRS Brain AnalyzIR toolbox (https://github.com/huppertt/nirs-toolbox),^115^ enables to use the systemic physiological signals in a GLM as additional regressors to remove the physiological influence from the fNIRS data. tCCA in combination with a GLM has been shown to be a particular promising approach for this task.^101^

Q: How does short-channel regression compare to SPA-fNIRS-based regression of physiological noise?

A: While classical fNIRS devices only measured with long SDSs, i.e., long-channels, newer ones also enable to measure at short SDSs, i.e., short-channels. While the long-channels contain information from the intracerebral and extracerebral compartment, short-channels probe the extracerebral compartment in particular. Short-channels should have a SDS of ∼0.8  cm in adults and 0.2 cm in term-age infants,^116^ should be located as close as 1.5 cm to the corresponding long-channels,^117^ and the number of short-channels should be optimized depending on the number of position of the long-channels. The heterogeneity of extracerebral hemodynamics needs to be considered too.^118^^,^^119^ Various methods how to use short-channels have been developed,^118^^,^^120^^–^^126^ relying all on regressing out the extracerebral information from the long-channels (short-channel regression). The methods improve the ability to measure changes related to NVC^98^^,^^118^^,^^126^^–^^129^ and increase the reproducibility of fNIRS measurements on the single-subject level.^130^

**Fig. 9:**
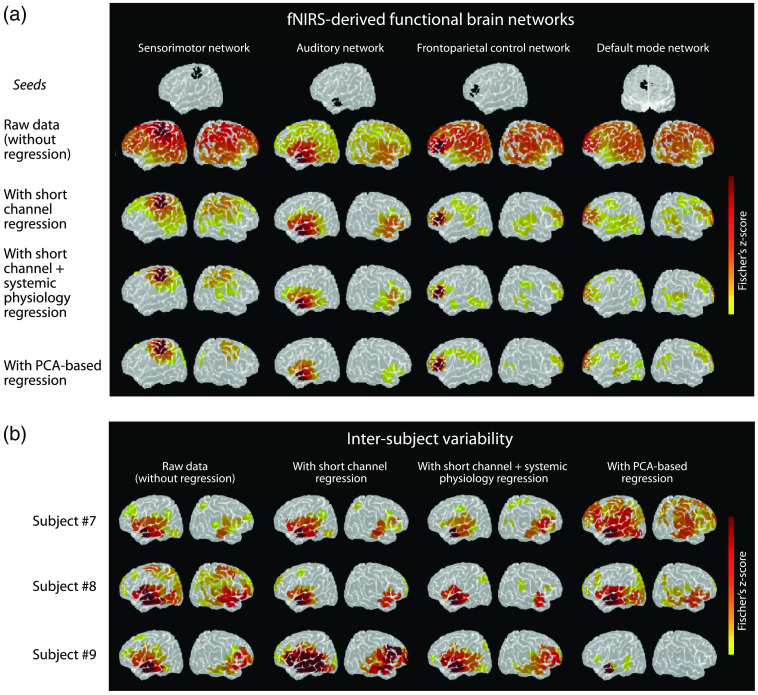
Comparison between different techniques to remove confounding physiological factors in resting-state fNIRS measurements. Shown are the findings of Abdalmalak et al.^99^ They investigated the impact of applying different physiological interference reduction methods on detecting resting-state functional brain networks. Depicted are the functional brain networks determined using raw data, with short-channel regression, with short-channel and systemic physiology (HR, MAP, and $P_{\mathrm{ET}}{\mathrm{CO}}_{2}$) regression, and with PCA-based regression. A whole-head fNIRS montage was used with 121 long-channels (SDS: 3 cm) and eight short-channels (SDS: 0.8 cm). (a) Group-averages and (b) single-subject variability (examples of three subjects; seed: auditory network). The single-subject analysis shows that there is a more distinct difference between the PCA-based regression and the short-channel and systemic physiology regression compared to the group-level analysis. The study shows the necessity and usefulness of the SPA-fNIRS to determine functional resting-state networks. Subfigures (a) and (b) modified from Abdalmalak et al.^99^

A comparison between short-channel regression, and SPA-fNIRS-based regression has been recently published by Abdalmalak et al.^99^ investigating the impact of both methods on the detection and quantification of functional resting-state brain networks. The authors concluded that both approaches are useful and necessary to detect the functional networks and that short-channels and systemic physiological signals “provided complementary information that could not be obtained by either regressor separately.” Figure 9 shows the impact of different regression methods of fNIRS resting-state functional connectivity measurements. The work of Abdalmalak et al. also showed that regressions based on principal component analysis (PCA) of long-channels are useful too. The resting-state networks detected were similar with PCA-based regression compared to regression with short-channels and systemic physiology. PCA-based regression seems to be therefore a good alternative to the SPA-fNIRS approach in case of resting-state measurements; however, the PCA method requires coverage of large areas of the head to ensure optimal capturing of the non-neuronally evoked changes in fNIRS signals, and the number of principal components to remove is arbitrary and is generally chosen so that the covariance to be regressed has a specific value (e.g., 80%).^99^^,^^131^ In a recent analysis of different fNIRS signal processing approaches to deal with physiological noise, PCA-based regression without short-channels has been found to be helpful, but regression with short-channels was superior.^132^

Short-channels and systemic physiological signals contain shared components, but also components unique to each. That the combination of short-channels and systemic physiological signals help to remove physiological noise in a GLM framework with fNIRS data from whole-head recordings has been shown recently.^98^ We expect that the number of such investigations will increase in the near future enabling one to determine when each method works best.

Q: Which aspects of SPA-NIRS still need to be further developed? And what are the open questions regarding the implementation and use of SPA-fNIRS?

A: Further developments of SPA-fNIRS should focus on hardware developments, e.g. the creation of integrated measurements devices that cover all relevant physiological signals along fNIRS measurements, as well as software developments, e.g. mathematical methods for SPA-fNIRS signal processing and analysis as well as software-implementations (ideally open-source) of the methods. With regard to applications of SPA-fNIRS, future studies need to explore the use of SPA-fNIRS for example in hyperscanning studies,^133^ in naturalistic environments,^7^ with children or in combination with other neurophysiological measurements, e.g., EEG. SPA-fNIRS will be an excellent approach to study the interplay between brain and body, supporting an integrated assessment of physiology in experimental studies.

Q: Will SPA-fNIRS be a new standard for fNIRS neuroimaging studies soon?

A: Giving the increasing awareness (i) that hemodynamic-based neuroimaging methods (fMRI and fNIRS) are influenced to a greater extent than previously acknowledged by factors not resulting from NVC^23^^,^^27^^,^^28^^,^^134^ and (ii) that there is a need to explore the link between the brain and the physiological state of the entire body (the embodied brain), we expect that the SPA-fNIRS approach will become increasingly important in the future and that it will be used more frequently. There will be more research on SPA-fNIRS. Whether and how SPA-fNIRS is implemented and used in an fNIRS study depends on the study design and other factors, such as the availability of the necessary devices for the physiological measurement, and to performing additional data analyses and the feasibility of SPA-fNIRS measurements. There is not yet enough data on the realization and added value of SPA-fNIRS measurements in children (neonates and infants), but we expect that such measurements are feasible and will also significantly improve fNIRS data interpretation. We are aware that SPA-fNIRS measurements may be difficult to perform in specific experimental settings, e.g., because the necessary additional sensors may interfere with the experiment, restrict the subject’s freedom of movement or cause unpleasant sensations due to the skin contact of the sensors. The necessity and feasibility of SPA-fNIRS measurements must therefore be evaluated individually for each experimental setting.

Recently, the first SPA-fNIRS hyperscanning studies were performed, showing the usefulness and potential of the addition of measuring systemic physiological signals along fNIRS neuroimaging to investigate the functional body-to-body body-to-brain and brain-to-brain coupling in interacting subjects.^135^^,^^136^ The term “SPA-fNIRS hyperscanning” was introduced for this methodology.^136^

## 3 Concluding Remarks and Future Perspectives

We provided an outlook why neuroimaging using fNIRS will profit tremendously from systemic physiological activity being measured simultaneously by employing SPA-fNIRS. This approach assist enabling to properly interpret fNIRS signals, i.e., by allowing one to distinguish fNIRS signal changes due to brain activity and systemic physiological activity. It opens new dimensions to explore the complex interplay between brain activity and body physiology.

SPA-fNIRS is a new approach with great promise for the future, in our opinion. Only a few studies are yet available that explored the full potential of SPA-fNIRS; a fact that we expect to change since more and more neuroscientists will realize the value of including changes in systemic physiology when brain imaging techniques based on hemodynamics (fNIRS and fMRI) are employed. This trend can currently also be witnessed in the fMRI domain, where fundamental discoveries show the significant impact of systemic physiology on the signals and that there is an intricate interplay between different drivers of cerebral hemodynamics.^31^^,^^33^^,^^137^

Since SPA-fNIRS will help regressing out physiological influences in the fNIRS data, it will increase the interpretability of fNIRS signals. This approach will contribute to solving the current reproducibility crisis in functional neuroimaging studies by reducing false-positive or negative results.^138^ Furthermore, SPA-fNIRS has a great potential for personalized neuroscience,^139^ i.e., the investigation of the individually specific neurosystemic functional connectivity. SPA-fNIRS has also a great potential to understand if the fNIRS-derived functional resting-state and task-positive networks represents more resting-state physiological networks or genuine NVC-associated neuronal networks. Since the interplay between these two types of networks is currently an open research question,^140^ SPA-fNIRS could possible provide novel answers to this question. In addition, the extension of the fNIRS hyperscanning approach^133^ to SPA-fNIRS hyperscanning, as recently demonstrated,^135^^,^^136^ will allow one to explore the physiological functional coupling between interacting subjects in a new way.

There are still many fundamental open questions regarding SPA-fNIRS, which will stimulate many new research projects. Among other things, we need to determine which systemic physiological parameters should be measured for different experimental paradigms and subject groups (e.g., adults, children, and neonates), which signal processing approaches are best suited for SPA-fNIRS data and what additional information can be extracted from SPA-fNIRS data. Further research is needed on all these and other questions —our outlook paper should also serve as an encouragement to pursue these questions.

However, based on the data and insights gained so far with SPA-fNIRS, we can already state that SPA-fNIRS has great potential to improve fNIRS neuroimaging (by reducing the impact of physiological noise on fNIRS data) and to make it possible to discover significant novel insights into the complex interplay between the brain and the body.

## Data and Code Availability Statement

There is no data or code that could be made available.
